# Incorporating gender, equity, and human rights into the action planning process: moving from rhetoric to action

**DOI:** 10.3402/gha.v9.30870

**Published:** 2016-09-06

**Authors:** Sanjeev Sridharan, Joanna Maplazi, Apurva Shirodkar, Emma Richardson, April Nakaima

**Affiliations:** The Evaluation Centre for Complex Health Interventions, St. Michael's Hospital, Toronto, Canada

**Keywords:** action planning, gender, equity, and human rights, theory-driven evaluation

## Abstract

**Background:**

Mainstreaming of gender, equity, and human rights (GER) is an important focus of the World Health Organization (WHO) and other UN organizations. This paper explores the role of action plans in mainstreaming GER. This paper is informed by a theory-driven evaluation lens.

**Design:**

A theory of change framework explored the following seven dimensions of how action plans can implement mainstreaming of GER: awareness of the foundations of GER; understanding of context; planning to impact GER; implementation for GER; monitoring, evaluation, and learning; planning for sustainability; agenda setting and buy-in. The seven dimensions were used to analyze the action plans. Reviewers also explored innovations within each of the action plans for the seven dimensions.

**Results:**

GER mainstreaming is more prominent in the foundation, background, and planning components of the plan but becomes less so along the theory of change including implementation; monitoring and evaluation; sustainability; and agenda setting and buy-in.

**Conclusions:**

Our analysis demonstrates that much more can be done to incorporate GER considerations into the action planning process. Nine specific recommendations are identified for WHO and other organizations. A theory-driven approach as described in the paper is potentially helpful for developing clarity by which action plans can help with mainstreaming GER considerations.

## Introduction

As part the growing recognition of gender, equity, and human rights (GER) in health, the World Health Organization (WHO) Director-General Margaret Chan stated that a goal of mainstreaming was ‘to achieve a WHO in which each staff member has the core value of gender, equity and human right in his/her DNA’. (1). Although mainstreaming has been the focus of a number of WHO publications, the ‘how to’ mainstream GER remains unclear. Mainstreaming at WHO can be defined as (2): ‘Institutional mainstreaming of equity, gender and human rights implies that WHO structures, procedures and mechanisms should enable and facilitate the development, implementation and monitoring of health programmes and plans that are gender-responsive, enhance equity and promote rights, both in WHO and in its technical support programmes’.

This paper explores whether such an aspiration is demonstrated in the action planning process at WHO. Fifteen action plans (10 global and 5 regional plans) are analyzed as part of this paper. Given that mainstreaming of GER is still new at WHO, this paper is intended to serve an exploratory, developmental purpose (3). In evaluation parlance, the theory of change (4) of mainstreaming GER through action plans needs to be developed. The primary goal of this paper is not an assessment of the action plans but rather a formative analysis to learn more about how the action plans can help with mainstreaming GER. The critical question addressed in this paper is: How can future action planning better incorporate GER considerations into the planning process?

The approach adopted in this paper is to analyze the 15 plans and describe the state of incorporation of GER into existing action plans both at the global and regional levels of WHO. The analysis serves to learn from the action plans – to develop knowledge of how best to mainstream GER.

The Evaluation Centre for Complex Health Interventions based in Toronto, Canada, was commissioned by the GER team at WHO to develop an e-learning tool for WHO managers on integration of GER into action plans. This project was commissioned as a result of WHO's interest in incorporating GER considerations into the action planning process. This paper does not discuss the specifics of the e-tool. As example, interviews with program leads were an integral part of developing feedback on the e-tool. We neither highlight the details of the e-tool nor the discussions with the WHO staff on their perspectives for the need of an e-tool. Instead, in this paper we explore how theory-driven evaluation (4) approaches can help in incorporating GER considerations into action planning.

A WHO action plan can be viewed as a commitment by WHO's Member States to take action with specific global or regional targets attached to it. The action plan provides a good setting for bringing the core components of GER in policies and programs at WHO. Action plans are a good instrument to mainstream GER into the DNA of WHO.

A theory of change describes the relationship between intervention activities, outputs, and short- and long-term outcomes (4). The insight in this paper is to view the action plan itself as an intervention. The analytical question explored in this paper is: What are the pathways by which action plans can impact short- and long-term outcomes related to mainstreaming?

Key aspects of action plans that make them useful for mainstreaming GER include:

- Action plans are endorsed by WHO governing bodies at global and regional levels.
- Through the governing bodies, WHO is required to report every few years on implementation of the action plan, collecting information also from Member States.
- WHO work plans for bienniums reflect operationalization of components of action plans, and/or overall technical assistance packages to support governments to deliver on creation of national action plans that reflect the regional/global ones. WHO work plans are monitored periodically.
- Often action plans for specific areas are reflected in the Country Cooperation Strategies and United Nations Development Action Framework (UNDAF) for countries, which also serve as mechanisms for framing WHO and government cooperation.
- Depending on the exact nature of the action plan, some may be more binding for Member States than others.

Important implications from the above discussion is that action plans can impact mainstreaming of GER through multiple mechanisms including: legitimacy (as action plans are endorsed by WHO governing bodies), accountability (through the governing bodies), and cohesion (the action plans provide a cohesive framework for action).

As a starting point, it will be useful to consider mainstreaming from GER perspectives. Some ideas of what mainstreaming GER means are discussed in the South-East Asia Regional Office of WHO website (5): mainstreaming GER can result in the ‘integration of core values’ and alignment of core values across UN organizations, enhanced collective effort, and increased literacy of staff on ‘values and skills in order to incorporate them in strategic planning’.

Much of the focus on mainstreaming has been on gender (6, 7). In 1997, the United Nations Economic and Social Council (8) defined gender mainstreaming as: ‘… the process of assessing the implications for women and men of any planned action, including legislation, policies or programmes, in all areas and at all levels. It is a strategy for making women's as well as men's concerns and experiences an integral dimension of the design, implementation, monitoring and evaluation of policies and programmes in all political, economic and societal spheres so that women and men benefit equally and inequality is not perpetuated’.

The International Labour Organization gender equality tool (9) reports on the far reaching nature of mainstreaming gender: ‘Mainstreaming is not about adding a “woman's component” or even a “gender equality component” into an existing activity. It goes beyond increasing women's participation; it means bringing the experience, knowledge, and interests of women and men to bear on the development agenda. It may entail identifying the need for changes in that agenda. It may require changes in goals, strategies, and actions so that both women and men can influence, participate in, and benefit from development processes. The goal of mainstreaming gender equality is thus the transformation of unequal social and institutional structures into equal and just structures for both men and women’.

From an equity lens, the perspectives focus on stakeholder engagement, institutional capacities, access, and multiple definitions of disadvantage. As example, consider the 2008 report titled ‘Mainstreaming Health Equity in the Development Agenda of African Countries’ (10): ‘Progress would require very strong political will both in the legislative and executive branches of government. Strong citizen and stakeholder engagement is important. It is equally important that health equity policies, objectives and goals in national development plans must be clearly defined. In addition, governments and their partners need to build and strengthen institutional capacity for health equity surveillance systems at the national and sub-national levels for effective monitoring of health equity goals and evaluation of the health equity impact of economic and social policies. Access – both nominal and effective access – to health facilities and services, especially for disadvantaged group has to be expanded and health services delivery needs to be professionalized in order to provide equitable access to health services to achieve good health outcomes’.

The human rights perspective brings a clearer focus on ‘instruments’, ‘standards’, and development of capacities. As example, Table 1 describes the implications of mainstreaming from a human rights lens (11). The human rights perspective also engages with legally binding obligations of the International Covenant on Economic, Social, and Cultural Rights (12). Article 12 of the Covenant recognizes ‘the right of everyone to the enjoyment of the highest attainable standard of physical and mental health’. Also the human rights perspective is complemented by publications from other UN institutions (13).

**Table 1:** Mainstreaming from a human rights perspective (10)

All programs of development cooperation, policies and technical assistance should further the realization of human rights as laid down in the Universal Declaration of Human Rights and other international human rights instruments. Human rights standards contained in, and principles derived from, the Universal Declaration of Human Rights and other international human rights instruments guide all development cooperation and programming in all sectors and in all phases of the programming process. Development cooperation contributes to the development of the capacities of ‘duty bearers’ to meet their obligations and/or of ‘rights-holders’ to claim their rights.

The literature also argues for the central role of planning in the mainstreaming process. As example, consider Tobing-Klein (14): ‘By doing so we have to keep in mind, that gender mainstreaming is not only about the implementation of measures to help women, but most importantly to mobilise all general policies and measures specifically for the purpose of achieving equality by actively and openly taking into account at the planning stage their possible effects on the respective situation of men and women (gender perspective)’.

The actual evidence of mainstreaming is a bit more checkered than what some of the above aspirations would suggest. As example, Table 2 describes some of the conclusions from a 2008 African Development Bank Group report titled ‘Mainstreaming Gender Equality: A Road to Results or A Road to Nowhere?’ (15).

**Table 2:** Conclusions from an evaluation of gender mainstreaming (12)

**Conclusion 1:** Leadership has not consistently supported the implementation of gender mainstreaming policy, resulting in what has been widely described as ‘policy evaporation’.
**Conclusion 2:** The absence of accountability and incentive systems to systematize the integration of gender equality across organizations and interventions has limited the achievement of results.
**Conclusion 3:** Financial and human resources have not been sufficient to enable effective mainstreaming of gender equality within donor organizations and interventions.
**Conclusion 4:** Many procedures and practices have been introduced following the adoption of new gender policies or strategies, but have been actively pursued for only a short period before gradually declining in use.
**Conclusion 5:** Results reporting and learning have been seriously challenged by inconsistent approaches to monitoring and evaluation of gender mainstreaming.

There is a need to move the focus of mainstreaming GER beyond aspirational rhetoric, towards understanding how concrete actions can lead towards mainstreaming GER. A key assumption we make in the approach adopted in this paper is to treat the action plan itself as an intervention. As noted above, the action plan is an instrument of change. We explore what it would take for the action plan to impact outcomes related to mainstreaming.

## Evaluation approach: data and methods

The approach to the analysis of GER in the action plans is informed by a theory-driven evaluation perspective (16–19). Theory-driven evaluation begins by exploring the pathways by which an intervention – in this case the action plan itself – can impact outcomes.

Intervention theories have a long journey. They begin in the heads of policy architects, pass into the hands of practitioners and managers, and (sometimes) into the hearts and minds of clients and patients. Depending on the initiative, different groups will be crucial to implementation; sometimes the flow from management to staff (and through its different levels) will be the vital link; at other times the participation of the ‘general public’ will be the key interchange. The critical upshot of this feature is that interventions carry not one, but several implicit mechanisms of action. The success of an intervention thus depends on the cumulative success of the entire sequence of these mechanisms as the programme unfolds. (20)

One advantage of taking a theory-driven lens is the recognition that mainstreaming GER cannot solely be aspirational. Thinking theoretically about interventions sheds lights on the individual steps in the long journey of an intervention having impact. A theory of change helps make the key assumptions explicit (21).

The question we explore is: What are key mechanisms of mainstreaming GER in action plans that lead towards implementation that can impact issues of gender, equity and human rights? Theory-driven consideration helps shed light on the key assumptions and support systems that are necessary for GER to be mainstreamed.

**Fig. 1:**
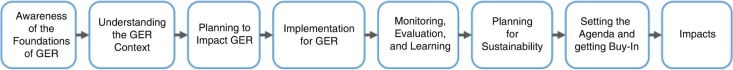
Simplified theory of change by which Action Plans can Impact Mainstreaming of GER 3.

A starting point of this theory-driven approach is developing an idealized theory of change (4) for how mainstreaming GER in the plans can impact outcomes. Although there are many pathways by which action planning can have an impact, the simplified theory of change below outlines seven specific dimensions of mainstreaming GER in an action plan (Fig. 1). The theory of change recognizes that action plans in and of themselves will not have an impact on GER mainstreaming; implementation, monitoring, learning, evaluation, agenda setting, and obtaining buy-in are perhaps other important processes in mainstreaming GER.

The analysis framework was developed both through a dialogue with WHO staff as well as a review of the relevant literature. One example of the literature that informed our work was the theory-driven evaluation literature (16–21). In this literature there is clarity on surfacing a theory of change by which action planning could lead to the mainstreaming of GER. This literature brings attention to not just treating the action plan as a product but also calls attention to the processes by which the action plan can impact mainstreaming. There is also an evaluation literature that focuses on planning for sustainability (22–25). This literature also informed the development of our instrument.

A 74-item scale was developed that spans the seven dimensions outlined in the theory of change in Fig. 1 (Table 3). Tables 4–10 in the results section describe each of the items used to measure the seven dimensions. We appreciate it is unrealistic to expect an action plan to consider all of these elements. However, as noted earlier, our interest was to highlight some of the challenges in the journey from action planning of GER and uptake.

**Table 3:** Strategic dimensions used in the analysis

Dimension	Explanations
Strategic foundations for addressing GER	This dimension interrogates if there is a recognition of core GER elements in the strategic foundations of the action plan. While the action plans spanned a range of topics, acknowledging relevant GER issues and principles of GER in the foundational elements of the action plan, including the goals and objectives, was seen as important for laying the groundwork for more specific action in the rest of the plan. Eight items were included as part of this dimension. Some of these items included: awareness of the importance addressing GER as part of the program's focus, goals and objectives related to GER, a roadmap in the plan of how it proposed to address GER issues, and a recognition of the importance of participation of affected/at-risk individuals in design of programs and policies.
Consideration of context of GER	This dimension explores if the action plan incorporates and analyzes information related to GER. The review of the literature found a number of resources that outline the processes to support the mainstreaming of gender and human rights, into planning and policy documents (19, 20). These resources discuss particular information and analyses that can be used as a program or policy is being developed. Therefore, a signal that some mainstreaming process took place in the development of the action plan is the inclusion of an assessment of the GER issues related to the topic of the action plan. Defining the GER problem was seen as a necessary precursor to addressing GER. Some of the items included an analysis of trends and key indicators related to GER, analysis of the legal and policy context, and recognition of the relationship between GER and the health system. There were 13 items as part of this dimension.
Planning to impact GER	This dimension interrogates the clarity and rigor of GER mainstreaming in the planning part of the action plan. This section of the review template sought to uncover a seriousness of purpose in the plan to meaningfully address GER issues. Action plans are unlikely to impact GER if there is no actual plan specified to do so. Therefore, items in this section included: clarification of processes and plans to reach the needs of key affected populations, issues of quality and culture sensitivities, incorporating knowledge of contexts into the planning process, role of intersectoral action in mainstreaming GER, and connections to universal health coverage.
Implementation considerations	This dimension seeks to explore the extent to which the action plan sets itself up to be implemented in such a way that GER issues could be impacted. There is an assumption that action planning processes that think through key implementation considerations as they are being developed will be better implemented, as some risks or barriers may have already been identified and influenced the plans. The items interrogate whether the action plan was likely to be realistic and implemented. A range of implementation considerations were included in the review template, including financial and human resources, infrastructure, information systems, leadership required, a process to adapt the plan to the national and local contexts, incorporation of communication mechanisms, and clarification of accountability processes for mainstreaming GER. This dimension consisted of 18 items.
Measurement, learning, and evaluation	This dimension seeks to identify whether the action plan had a clear vision of what progress on GER, as it relates to its specific topic, looks like. This is seen as a significant indicator that the action plan has been designed with the intention of impacting GER and sets itself up to be held accountable for GER outcomes. This dimension consisted of 12 items, including defining progress for mainstreaming GER, definitions of measurable performance standards, systems and processes to monitor progress on GER, clarity on responsibilities for monitoring and evaluation of GER, a process of taking ownership of targets, attention to data quality issues, and processes for sharing information.
Planning for sustainability	Ultimately, mainstreaming is going to be a long-term process. Considerations for how action and progress on GER will be sustained need to be incorporated into the action planning process. This dimension consisted of four items, including paying attention to long-term organizational capacities, training and capacity needs, and identification of individual capacities needed for mainstreaming GER.
Agenda setting and buy-in	Finally, mainstreaming and impact on GER outcomes is unlikely to occur if the ideas are confined just to the action plan. There needs to be an explicit process of agenda setting and building buy-in for the action plan. This dimension consisted of two items on getting GER on the agenda of key stakeholders, and raising the salience of GER.

GER, gender, equity, and human rights.

**Table 4:** Items and analysis of items related to awareness of the foundations of GER

	Pattern of responses (number of plans in parenthesis)	
	
Items	% Yes	% No
Clear awareness of the importance to address GER issues as a step to address health issue	80 (12)	20 (3)
Inclusion of specific goals related to mainstreaming GER	40 (6)	60 (9)
Defined objective for above goals^a^	40 (6)	27 (4)
Inclusion of a road map/theory of change to mainstream GER	13 (2)	87 (13)
Identified actions intended to disrupt underlying causes of GER issues^a^	47 (7)	47 (7)
Proposal of downstream actions related to GER	33 (5)	67 (10)
Recognition of the importance and promotion of the participation of affected/at-risk individuals in design of policies, programs	80 (12)	20 (3)
Identification of international human rights treaties or conventions	40 (6)	60 (9)

a Some plans had missing cases as reviewers had the opportunity to leave an item blank if the details were unclear.

GER, gender, equity, and human rights.

**Table 5:** Items and analysis of items related to the understanding of GER context

	Pattern of responses (number of plans in parenthesis)	
	
Items	% Yes	% No
Analysis of trends and key indicators related to GER	40 (6)	60 (9)
Inclusion of disaggregated data by gender or income	53 (8)	47 (7)
Inclusion of disaggregated data by race, ability, age, language, and/or sexual orientation	33 (5)	67 (10)
Analysis of spatial distribution of key indicators of health	80 (12)	20 (3)
Analysis of the variations in existing services or service delivery by gender and/or other equity stratifiers	40 (6)	60 (9)
Inclusion of an analysis of health needs	47 (7)	53 (8)
Utilization of specialized tools or resources to assess needs^a^	7 (1)	87 (13)
Discussion of the needs of populations experiencing humanitarian crises, natural disasters or conflict	53 (8)	47 (7)
Discussion of the legal and policy context	73 (11)	27 (4)
Identification of groups at the lowest end of the health gradient	53 (8)	47 (7)
Acknowledgement of the inverse care law	60 (9)	40 (6)
Identification of health needs beyond physical health, including mental, emotional, social, and spiritual health and wellness	40 (6)	60 (9)
Recognition the GER problems are exacerbated by weak health and social protection systems	40 (6)	60 (9)

a Some plans had missing cases as reviewers had the opportunity to leave an item blank if the details were unclear.

GER, gender, equity, and human rights.

**Table 6:** Items and analysis of items related to planning

	Pattern of responses (number of plans in parenthesis)	
	
Items	% Yes	% No
Clear outline of plans, processes, or systems to reach and address the needs of key affected and at-risk populations	60 (9)	40 (6)
Connection of plans that relate to mainstreaming GER to the targets identified in Section A^a^	20 (3)	73 (11)
Identification of specific barriers to ensuring available and accessible services to groups at the lowest end of the health gradient^a^	73 (11)	20 (3)
Outline a plan to address issues of availability or access to groups at the lowest end of the health gradient^a^	60 (9)	33 (5)
Identification of specific actions to address quality of health services and services related to the determinants of health^a^	53 (8)	40 (6)
Incorporation of cultural sensitivities and language needs	47 (7)	53 (8)
Inclusion of evidence or knowledge of what actions are likely to be successful for specific groups^a^ (i.e. ‘what can work for whom’)	0 (0)	93 (14)
Discussion of the settings and contexts (laws/ policies) necessary for proposed actions to be effective	73 (11)	27 (4)
Proposal for intersectoral action to address GER	80 (12)	20 (3)
Discussion of an explicit plan to provide a comprehensive set of services	33 (5)	67 (10)
Promotion of an assets-based approach to mainstreaming GER	27 (4)	73 (11)
Promotion of an health-in-all policies agenda	53 (8)	47 (7)
Inclusion/discussion of Universal Health Coverage	60 (9)	40 (6)
Identification of actions to address health system building blocks (health governance, financing, workforce, medical products and technologies, and information and research)	87 (13)	13 (2)
Discussion of how to communicate effectively with groups at the lowest end of the health gradient (public information/education/prevention campaigns)	33 (5)	67 (10)
Recognition of gender equality as one precondition for an effective plan	0 (0)	100 (15)
Recognition that resource constraints may prevent the full realization of the right to health^a^	40 (6)	53 (8)

a Some plans had missing cases as reviewers had the opportunity to leave an item blank if the details were unclear.

GER, gender, equity, and human rights.

**Table 7:** Items and analysis of items related to implementation

	Pattern of responses (number of plans in parenthesis)	
	
Items	% Yes	% No
Discussion of how GER will be integrated or mainstreamed	7 (1)	93 (14)
Identification of processes and procedures to support implementation of mainstreaming GER	7 (1)	93 (14)
Discussion of the financial resources needed to support implementation of mainstreaming GER	20 (3)	80 (12)
Identification of human resources needed to support implementation of mainstreaming GER	7 (1)	93 (14)
Discussion of the infrastructure needed to support implementation of integrating GER considerations	7 (1)	93 (14)
Discussion of information systems and information governance needed to support implementation of the action plan	60 (9)	40 (6)
Identification of the leadership required to support implementation	47 (7)	53 (8)
Discussion of the organizational capacities and capabilities needed to support implementation	47 (7)	53 (8)
Discussion of how the quality of implementation related to GER considerations will be addressed	0 (0)	100 (15)
Identification of the need to adapt implementation to national and local contexts	93 (14)	7 (1)
Discussion of processes to adapt based on feedback from stakeholders and participants	0 (0)	100 (15)
Discussion of how monitoring of services will vary based on groups served	13 (2)	87 (13)
Specification of communication mechanisms for implementation	20 (3)	80 (12)
Creation of processes or systems to target and reach individuals with greater needs	40 (6)	60 (9)
Outlines specific action steps to address the needs of affected and high risk populations	40 (6)	60 (9)
Proposal of action related to affordability and financial access to prevention and treatment services	60 (9)	40 (6)
Proposal of action to prevent catastrophic health expenditures	40 (6)	60 (9)
Specification of accountability processes and mechanisms related to mainstreaming GER	0 (0)	100 (15)

GER, gender, equity, and human rights.

**Table 8:** Items and analysis of items related to monitoring, evaluation, and learning

	Pattern of responses (number of plans in parenthesis)	
	
Items	% Yes	% No
Discussion of what progress related to mainstreaming GER looks like	7 (1)	93 (14)
Definition of measurable performance standards for identified actions related to GER	0 (0)	100 (15)
Outlines a system to monitor performance against GER performance standards	0 (0)	100 (15)
Outlines processes for monitoring GER data	27 (4)	73 (11)
Inclusion of plans to conduct evaluations to measure progress in mainstreaming GER	0 (0)	100 (15)
Identification of who will be responsible for monitoring and evaluation	47 (7)	53 (8)
Discussion of how baseline data will be gathered^a^	33 (5)	60 (9)
Outlines a plan to analyze results by key sub-groups	20 (3)	80 (12)
Outlines a plan to analyze distributional impacts of actions	20 (3)	80 (12)
Proposal of monitoring access, uptake, and completion rates to identify differences between population groups	0 (0)	100 (15)
Encouragement of ownership of targets related to GER through effective performance management	7 (1)	93 (14)
Identification of data quality issues and proposal of strategies to improve data quality	53 (8)	47 (7)
Specification of how performance and progress related to mainstreaming GER will be shared with key stakeholders	7 (1)	93 (14)

a Some plans had missing cases as reviewers had the opportunity to leave an item blank if the details were unclear.

GER, gender, equity, and human rights.

**Table 9:** Items and analysis of items related to planning for sustainability

	Pattern of responses (number of plans in parenthesis)	
	
Items	% Yes	% No
Discussion of long-term organization capacities needed to deliver on recommended actions for mainstreaming GER	0 (0)	100 (15)
Identification of training and capacity building needs of key personnel to support individual competencies related to GER	0 (0)	100 (15)
Identification of processes to ensure organizations fulfill their commitments and responsibilities	13 (2)	87 (13)
Identification of multiple organizations that can lead in bringing GER perspective	7 (1)	93 (14)

GER, gender, equity, and human rights.

**Table 10:** Items and analysis of items related to agenda setting and getting buy-in

	Pattern of responses (number of plans in parenthesis)	
	
Items	% Yes	% No
Discussion of specific actions to place GER issues on the agendas of key stakeholders	7 (1)	93 (14)
Discussion of specific actions to raise the salience of GER issues and the promotion of buy-in	7 (1)	93 (14)

GER, gender, equity, and human rights.

### Sampling and methodology

The selection of plans was made by the WHO program officers in consultation with the GER cluster focal points; the sampling was purposive and chosen to represent a diversity of programmatic areas and included 10 global and 5 regional plans. Such a sampling strategy was consistent with the learning and exploratory focus of the evaluation. The plans are all freely available on the worldwide web; we have chosen not to identify the plans in this paper as we are keen not to assess individual plans but learn broader lessons.

Three reviewers completed the review template for 15 action plans – 5 action plans per reviewer. The reviewers were staff of the Evaluation Centre for Complex Health Interventions. They were evaluators with graduate degrees in Public Health/Health Sciences and with training in evaluation methods of complex health interventions. Each of the 74 items in the review template consisted of a series of ‘yes–no’ questions. If the answer was ‘yes’ to a question, the reviewer was prompted to describe the issue in greater detail. Each of the dimensions included an open question on any noteworthy or exemplary aspect of the action plan. Reviewers also had the opportunity to leave an item blank if the details were unclear. The reviewers were given the following instructions, ‘while the template mentions gender, equity and human rights, you are encouraged whenever possible to identify and explain these three elements independently in your response. Please note that all of the plans are focused on specific health problems – and not in themselves focused on GER as a separate dimension from the problem being addressed. The interest in the template is to explore if GER considerations are embedded in addressing the health problem’.

The review of each of the action plans was exhaustive, with completion of the review template taking approximately 2 days for each action plan. After the first review of the action plans was completed, reviewers were asked to look for innovations within each of the plans for each of the dimensions in a second review. Reviewers met regularly and iteratively to discuss each other's work and align the ratings process.

## Results

In general, the average rating falls as the dimensions progress along the program logic from ‘awareness’ to ‘setting the agenda’. As the dimensions are based upon the progression outlined in the theory of change, this means that GER mainstreaming is more prominent in the foundational, background, and planning components of the plan, but becomes less so for components further along the theory of change, including implementation, monitoring and evaluation, sustainability, and agenda setting and buy-in. This result is consistent with our experience with similar evaluations of strategic planning processes (25); there is a need to more explicitly incorporate implementation, monitoring and evaluation, agenda setting, and sustainability criteria in the planning process itself.

Tables (4–10) show results corresponding to the seven dimensions. Please note that some items had a few missing cases as the reviewers were unable to classify a plan if the details pertaining to that item were missing.

In the first dimension, *Strategic Foundations for Addressing GER*, most of the plans (80%) demonstrated some awareness of the importance of addressing GER issues as a step to addressing health issues and recognized the importance of participation of affected and at-risk individuals; however, far fewer (40%) identified a specific goal or objective related to GER.

The most common attributes of the action plans related to the second dimension, *GER Background and Context*, was an analysis of the spatial distribution of key indicators of health (80%) and a discussion of the legal and policy context (73%). About half the plans (53%) included disaggregated data by gender or income and identified groups at the lowest end of the health gradient. Only one plan (7%) used specialized tools or resources to assess needs.

The third dimension, *Planning to Impact GER*, had the highest mean rating of all the dimensions. Most of the plans (83%) identified actions to address health system building blocks (i.e. health governance, financing, workforce, medical products and technologies, and information and research). A majority (80%) also proposed intersectoral action to address GER. No action plans included information on what actions would be likely to be successful for specific groups or recognized gender equality as a precondition for an effective plan.

The average rating begins to decline with the fourth dimension, *Implementation Considerations*. Although almost all plans (93%) identified the need to adapt implementation to national and local contexts, for 7 of the 18 items in this dimension, only one or no action plans reflected a response of ‘yes’ on the item. This includes discussing processes to adapt implementation based on feedback from stakeholders and participants, and identifying processes, procedures, human resources, and infrastructure to support implementation of mainstreaming GER.

In the *Monitoring*, *Learning*, *and Evaluation Dimension*, about half the action plans identified data quality issues (53%) and identified who would be responsible for monitoring and evaluation (47%). However, most action plans rated very poorly for items focused specifically related to GER, for example, proposing monitoring access, uptake, and completion rates to identify differences between population groups.

In the final two dimensions, very few plans had a response of ‘yes’ to any of the items. Two action plans (13%) identified processes to ensure that organizations fulfill their commitments and responsibilities in the *Planning for Sustainability* dimension. For the two items in the *Agenda Setting and Buy-In* dimension, only one action plan responded positively to each item.

## Discussion

We stress that our interest in this paper has been to explore how a theory-driven perspective can help clarify the long journey from action planning to mainstreaming GER. This analysis has found that much work remains in incorporating GER considerations into the action planning process. A specific implication of our work is that the process of developing action plans needs guidance on how best GER considerations can be incorporated into the variety of WHO program areas. One specific implication of thinking theoretically is that when it comes to mainstreaming GER, grand visioning is not enough; attention needs to be paid to issues of implementation and sustainability if the journey from idea to reality needs to be completed. In this section we discuss recommendations from our work for future action planning processes that attempt to mainstream GER. Much of our focus is on the role of WHO but we think our findings are also relevant to other international, national, or regional organizations.

### Recommendation 1: Clarify process of integrating GER considerations into target setting in diverse program areas

There needs to be greater clarity how GER considerations can be integrated within the program targets across the diverse program areas (26). Most plans did not incorporate GER ideas into the targets across the program areas. There is a need for better examples how GER considerations can be integrated within the specific program areas. There is a need for leadership from both within WHO and other agencies interested in mainstreaming GER on leading a dialogue on providing examples of how GER-related considerations can be integrated in target setting within multiple program areas (both within WHO and other organizations).

### Recommendation 2: Promote applications of theories of change for mainstreaming GER

Most actions plans did not have either an implicit or an explicit theory of change (3) of how their program activities could impact GER-related outcomes. Theories of change need to identify the role of both concrete downstream and upstream actions that could lead to mainstreaming GER in a variety of action areas. One specific recommendation for organizations like WHO (or other international organizations such as UN Women) is to commission reviews of examples of theories of change of mainstreaming GER. Given the wide variety of program areas, it is important to describe whether the theories of change for mainstreaming need to differ across different program areas (and also provide examples of theories of change of mainstreaming GER within the program areas). Additionally such a review can also include interviews with planners on how best to incorporate GER considerations into the action planning process. Such interviews can also help identify ‘leverage points’ for concrete actions for mainstreaming GER within the various program areas. Such a distillation of developing theories of change of how GER needs to be shared widely with WHO program leads and staff and their feedback incorporated into any future guidance on what a theory of change means for mainstreaming GER into the diverse program areas.

### Recommendation 3: Promote the use of disaggregated data

A third area in which international organizations such as WHO could help is to promote mainstreaming of GER into action planning is by providing actual examples of the use of disaggregated data by specific equity stratifiers (such as gender) in the action planning process (27). Although there is appreciation of the use of disaggregated data in the program areas (28), how such disaggregated data can inform concrete actions for addressing GER concerns in a variety of program areas needs to be made more explicit.

### Recommendation 4: Promote the use of tools to understand the unmet needs related to GER

Addressing problems of equity and gender also implies identifying and understanding the nature of unmet needs (29). There is a need for tools that could help different groups understand issues of unmet need (most plans did not explicitly discuss the use of such tools to understand unmet need). We think there needs to be leadership from organizations like WHO to spread knowledge of how tools can be used to identify unmet needs in the action planning process.

### Recommendation 5: Incorporate knowledge of contexts and mechanisms into mainstreaming GER

The mechanisms by which GER considerations can be mainstreamed might differ across multiple contexts, including multiple program areas. Addressing problems of inequities often requires knowledge of what works for whom and under what circumstances (16). Most action plans did not discuss different strategies for addressing GER issues across multiple contexts. One concrete recommendation for international organizations like WHO would be to commission a realist synthesis (17, 20) in multiple program areas that can help develop knowledge of how mainstreaming can work across multiple contexts. A realist synthesis explores the contexts and mechanisms that are necessary for an intervention to work. By focusing on contexts and mechanisms, it identifies the conditions under which interventions are likely to work (17).

### Recommendation 6: Clarify what it takes to implement GER in a variety of program areas

Very few plans discuss the financial, human resources, and infrastructure needed to mainstream GER. This is to be expected, given that the focus of the action plans is not directly about GER – rather they need to incorporate GER into the various program areas. Once again, leadership from WHO is needed to commission reviews and case studies of mainstreaming experiences to explore the types of structures/processes and the supporting infrastructures that have helped with the implementation of the mainstreaming of GER.

### Recommendation 7: Clarify what progress in addressing GER actually means. Examples of accountability processes and mechanisms for mainstreaming GER need to be shared widely

There were very few examples in the action plans which demonstrated knowledge of how to measure progress in mainstreaming from a GER lens. None of the plans had any clarity on the accountability processes and mechanisms related to mainstreaming GER. Once again we think there is a need to commission reviews or conduct case studies of mainstreaming GER on what are progress markers and performance standards for identifying actions related to GER. Similarly, it will also be useful to identify examples of exemplary systems that have been developed in organizations either inside or outside WHO to monitor performance of mainstreaming GER.

### Recommendation 8: Clarify what ownership of mainstreaming GER targets means

As part of the monitoring and evaluation efforts there needs to be explicit clarity of how the different claim holders and duty bearers can take ownership of the GER targets. Although we recognize that action planning often focuses primarily on the planning aspects, we think it is important for the action planning process to more clearly identify processes and procedures to support implementation in mainstreaming GER as well as the role of different stakeholders in ‘owning’ the GER targets.

### Recommendation 9: Greater attention needs to be paid to processes for sustaining and promoting the mainstreaming of GER

Future action planning process needs to pay closer attention to the long-term organizational capacities and individual capacities and competencies that are needed to deliver in a sustainable manner on recommended actions for mainstreaming GER (21). None of the action plans analyzed had incorporated ideas of sustaining or planning to build buy-in for mainstreaming GER. Studies are needed for examples of exemplary jobs in raising the salience of GER, incorporating ideas of sustaining mainstreaming GER, and placing GER on the agenda of stakeholders.

### Limitations

We appreciate that the analysis in this paper has limitations, and we reiterate that the analysis is intended to be exploratory.

- A number of the items considered across the seven dimensions were intended to be explored in a spirit of brainstorming and we appreciate that not all of the items related to the seven dimensions are substantively relevant to the wide variety of program areas.
- Additionally our theory of change is only intended to be a simplified version of the complex process by which action planning can help with mainstreaming GER. We think there is need for much research on the processes by which action plans can contribute to mainstreaming. Given the ‘nascent’ state of the field that connects action planning to GER, our analysis is intended to be developmental and raise questions on the steps by which action planning can aid with mainstreaming. Future work needs to explore the different pathways by which action plans can have influence at the global, regional, and country levels.
- We have applied a theory of change approach to evaluate mainstreaming of GER in the action plans. There are a wide range of evaluation approaches that can be applied to evaluate action plans. We hope that this paper also encourages the applications of other evaluation approaches to mainstreaming of GER in the action plans.
- It is also important to reiterate that the analysis was not meant to be an assessment of the action plans. Rather, we view the analysis as a means of developing knowledge of the different steps needed to be made in the long journey from developing action plans to mainstreaming GER. Mainstreaming is a political process and we appreciate that the leverage of an action plan may be limited in impacting mainstreaming on its own; a number of other factors come into play in the mainstreaming process. However, our analysis has demonstrated that more can be done to incorporate GER considerations into the DNA of WHO.
- Our original plan was that multiple raters would read the 15 plans and we could compare the reliability across the different raters; however, this was not feasible as the analysis of each of the plans using the comprehensive set of dimensions turned out to be very involved. Our approach in the analysis for this paper was to have raters meet often to discuss individual aspects of plans and when in doubt to raise issues in team meetings.
- Our analysis essentially provides a top-down view of mainstreaming. This was, of course, necessary as we were analyzing plans at the global and regional levels. We appreciate that there are a number of other pathways/instruments by which mainstreaming can occur. Action plans are perhaps only one instrument in the mainstreaming process.
- An additional limitation is that our focus primarily has been on the role of single international organization (WHO) in the mainstreaming process. Although this is a limitation we think that the learning from this analysis also applies to other organizations.
- Space considerations prevent us from discussing the innovations that we found in individual plans. We are writing a separate paper identifying concrete examples of innovative ideas where action plans could help with mainstreaming GER.

## Conclusion

Our analysis demonstrates that more can be done to clarify how GER can be incorporated into the action planning process. Program areas vary and we appreciate that different programs might need different guidance to incorporate GER considerations into their action plans. We think a theory-driven approach as described in this paper is potentially helpful in developing clarity by which action plans can help with mainstreaming GER.

## Paper context

There is an interest within the World Health Organization to instill in their staff and in their programming core values of gender, equity, and human rights. The WHO action plan provides a good instrument to mainstream thinking into WHO programming. This paper describes how a theory-driven evaluation lens is helpful in potentially developing clarity and specificity by which WHO can mainstream GER. Nine specific recommendations are identified for WHO and other organizations to mainstream GER.
